# Treatment of aortic arch and left subclavian artery aneurysms in two stages using combined surgical approaches: case report

**DOI:** 10.1590/1677-5449.202401272

**Published:** 2025-04-07

**Authors:** Josué Song Der Wu, Lucas Porto Maurity Dias, Giuliano Giová Volpiani, Valter Castelli, Vanessa Prado dos Santos, Luiz Eduardo Meucci Pereira Nogueira, Walkíria Hueb Bernardi, Roberto Augusto Caffaro

**Affiliations:** 1 Santa Casa de São Paulo, Faculdade de Ciências Médicas, São Paulo, SP, Brasil.; 2 Irmandade da Santa Casa de Misericórdia de São Paulo – ISCMSP, São Paulo, SP, Brasil.; 3 Universidade Federal da Bahia – UFBA, Faculdade de Medicina da Bahia, Salvador, BA, Brasil.

**Keywords:** aortic arch, endovascular aneurysm repair, vascular surgical procedures

## Abstract

Aortic arch aneurysms are often asymptomatic, being diagnosed incidentally in tests such as computed tomography, magnetic resonance imaging, or echocardiogram. Conventional treatment involves thoracotomy surgery, although treatment can also be performed using endovascular techniques. This article presents a case report of a complex aneurysm of the aortic arch with involvement of the left subclavian artery. Treatment was initiated with debranching of the supra-aortic trunks by carotid-carotid and carotid-subclavian bypasses, followed by ligation of the common carotid and left subclavian arteries. A second procedure was then needed to construct a surgical vascular conduit using a Dacron graft to obtain access to the aortic arch for the stent graft delivery device, due to the narrow caliber of the external femoral and iliac arteries. This report illustrates a satisfactory outcome in a case of aortic arch aneurysm with complex anatomy.

## INTRODUCTION

Aortic aneurysms were related to the deaths of 172,427 people globally in 2019, equating to an 82.1% increase compared to 1990 (94,698 deaths).^1^ Despite the increase in absolute terms, the standardized mortality for age attributable to aortic aneurysms per 100,000 inhabitants actually followed a falling trend (17.9%), dropping from 2.70/100,000 inhabitants in 1990 to 2.21/100,000 inhabitants in 2019.^1^

Aneurysmal disease of the aorta is defined as a localized dilation involving all of the layers of the vessel wall and the abdominal aorta is the segment most often involved.^2^ Factors associated with greater risk of growth of aortic aneurysms include age, female sex, chronic obstructive pulmonary disease, arterial hypertension, family history, and aneurysm diameter.^3^ The literature shows that the initial diameter of the aneurysm influences its growth rate, in that larger aneurysms grow more rapidly.^3^ Aneurysms of the aortic arch are rare, are generally asymptomatic, and are challenging to diagnose and treat.^2^ The importance of the supra-aortic trunks and their major branches and the convexity of the aortic arch add significant complexity to treatment options for diseases involving the aortic arch.^2^

The natural history of aneurysms of the thoracic aorta and aortic arch presents as progressive increase vessel diameter, which should be monitored using imaging methods to decide on when to indicate surgery. The American Heart Association guidelines suggest that several different factors should be considered to determine whether surgery is indicated for aneurysms of the thoracic aorta, including diameter, growth rate, a detailed case assessment, diagnosis of diseases associated with dilation of the aorta, such as Marfan Syndrome, the experience of the team, and the proportional dimensions of the aorta, taking patient height into consideration.^4^ Surgical treatment of aneurysms of the thoracic aorta can be conducted using conventional surgery or endovascular techniques. For conventional surgery, a median sternotomy is needed to access the aortic arch to substitute the dilated portion of the aorta with a vascular prosthetic graft.^3^ The alternative, the endovascular technique, is less invasive and is performed using fluoroscopy to guide the procedure, which is performed via bilateral dissection or puncture of the common femoral arteries, with navigation using guide wires, and deployment of an endograft to line the aneurysmal aorta internally, excluding the aneurysm. To obtain adequate access for the endograft device, this technique generally requires the femoral and iliac to have a minimum caliber of 8 mm. Considering that conventional repair of aneurysms of the aortic arch needs flow to the supra-aortic trunks to be blocked, endovascular techniques have offered new solutions and alternative options for treatment of diseases of the aortic arch.^3^

In aortic diseases, the important decisions on whether intervention is indicated, the ideal type of surgical repair, and the choice of open surgical and/or endovascular options must be studied in great detail and decision-making should be shared.^4^ In this case report, we describe endovascular repair of an aneurysm of the aortic arch that required surgical construction of a prosthetic Dacron conduit to enable device access. This study was approved by the Research Ethics Committee at the Santa Casa de Misericórdia de São Paulo (Ethics Appraisal Submission Certificate: 80949724.9.0000.5479; decision number: 6.978.599).

## CASE DESCRIPTION

A 43-year-old man, a Bolivian national and resident, with hypertension and a smoking habit (25 pack years), was admitted to the emergency room at the Santa Casa de Misericórdia de São Paulo with retrosternal chest pain radiating to the back with onset around 1 year previously. He had sought medical attention due to exacerbation of the pain in the days leading up to presentation. He reported that 1 year previously he had undergone routine occupational tests including a chest X-ray that showed an enlarged mediastinum, which was investigated and diagnosed as aneurysmal disease of the aorta. No abnormalities were found during the physical examination.

**Figure 1:**
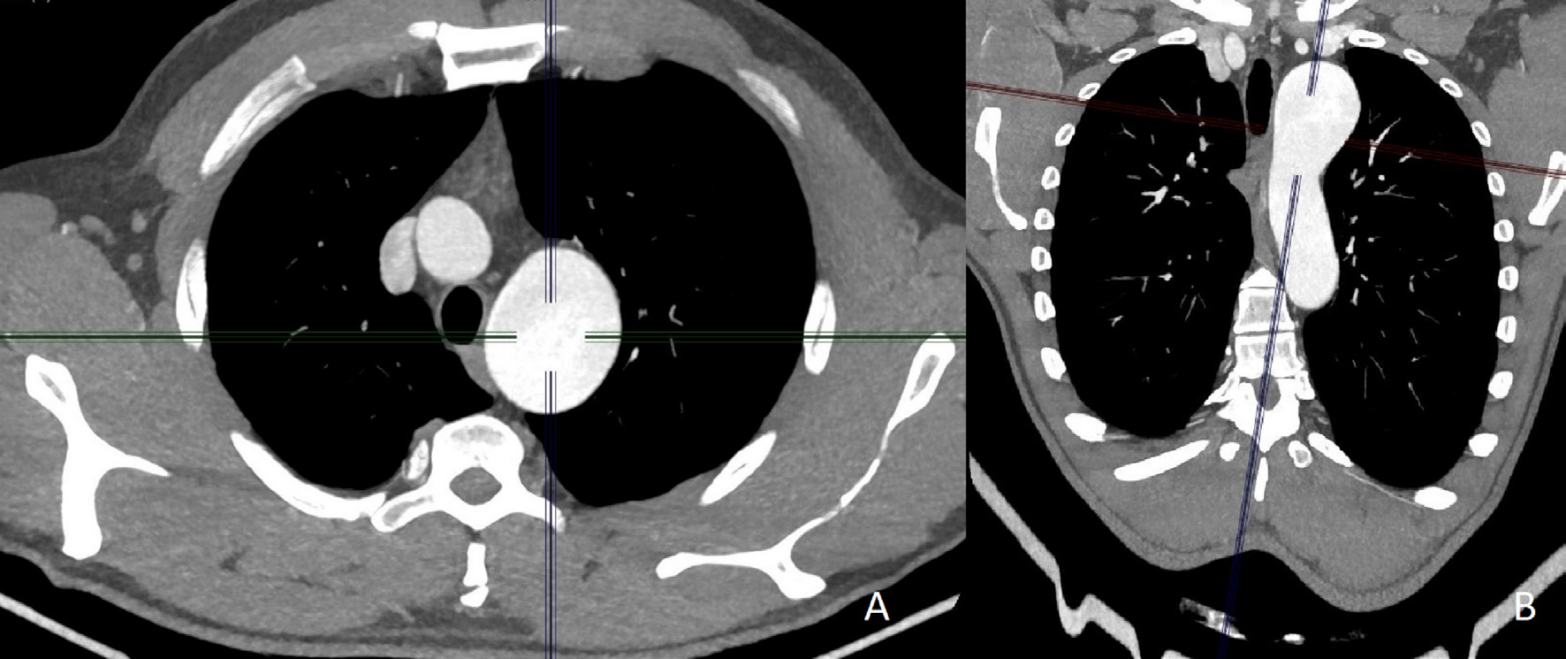
**A and B)** Transverse images from preoperative angiotomography of the aorta, showing the aortic arch aneurysm with left subclavian artery involvement, with a maximum cross-sectional diameter of 4.2 x 4.1 cm.

**Figure 2:**
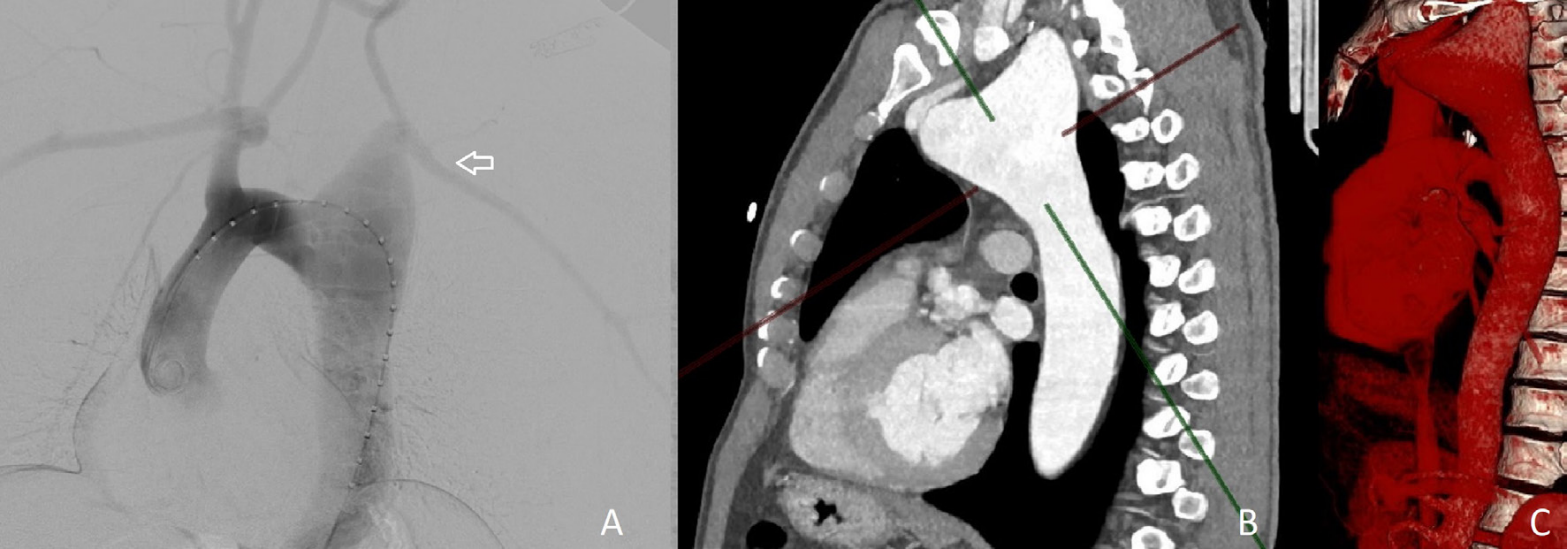
**A)** Digital subtraction angiography image and **B** and **C)** angiotomography showing aneurysmal dilation of the aortic arch and the left subclavian artery (arrow).

Angiotomography of the aorta showed a large aneurysm at the origin of the left subclavian artery and the distal portion of the aortic arch (Figure 1). The maximum cross-sectional diameter of the subclavian artery was 4.2 x 4.1 cm, with a length of approximately 4.2 cm (Figure 2). The patient was asymptomatic, and an imaging exam with contrast found no signs of rupture or imminent rupture. The management option chosen was therefore planned surgical repair of the aneurysm in stages. Detailed analysis of the dimensions and anatomy of the aneurysmal dilations showed that it would be necessary to ligate the left common carotid and subclavian arteries to obtain a proximal landing site that would be adequate to anchor the stent graft. Therefore, carotid-carotid and left carotid-subclavian bypass was performed with 6 mm wired expanded polytetrafluoroethylene (PTFE) graft to enable endovascular repair of the aortic arch aneurysm at a later date. The interval between the two procedures was 20 days and both were performed without intercurrent conditions.

For the first procedure, a longitudinal right cervicotomy was performed to access the common carotid artery and the same procedure was mirrored on the left. Additionally, an oblique left supraclavicular incision was made to identify and isolate the left subclavian artery, which was considerably dilated. Next, a carotid-carotid and left carotid-subclavian bypass was constructed using a PTFE prosthesis with a diameter of 6 mm, to maintain flow in the left subclavian and carotid arteries, followed by proximal ligation of the left subclavian artery and left common carotid, preventing filling of the aneurysm sac by back-flow and averting a future type 2 endoleak. Finally, the surgical site was inspected to guarantee hemostasis, drained, and sutured.

**Figure 3:**
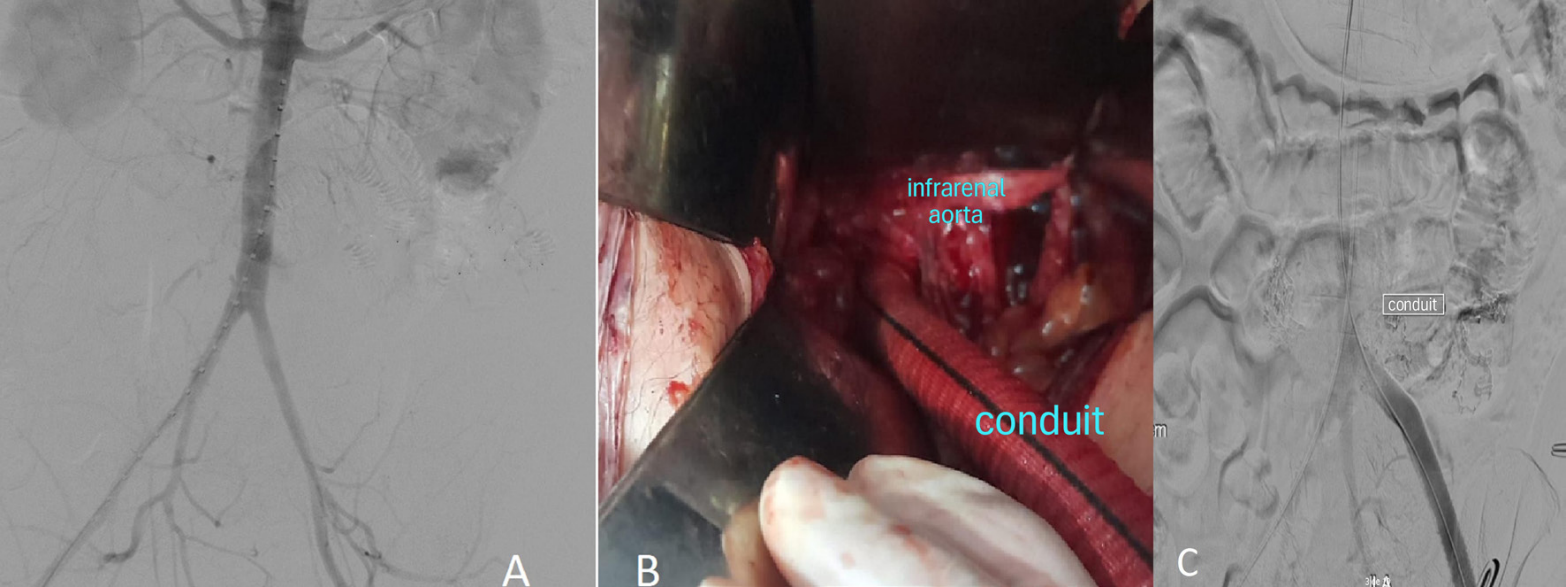
**A)** Angiography image showing the aortoiliac territory. **B)** Intraoperative image showing end-to-side anastomosis of the vascular conduit, constructed from an 11 mm straight Dacron graft, to the infrarenal aorta to provide left retroperitoneal access for the device. **C)** Arteriography showing the conduit constructed to provide access to the aortic arch for the endograft device.

**Figure 4:**
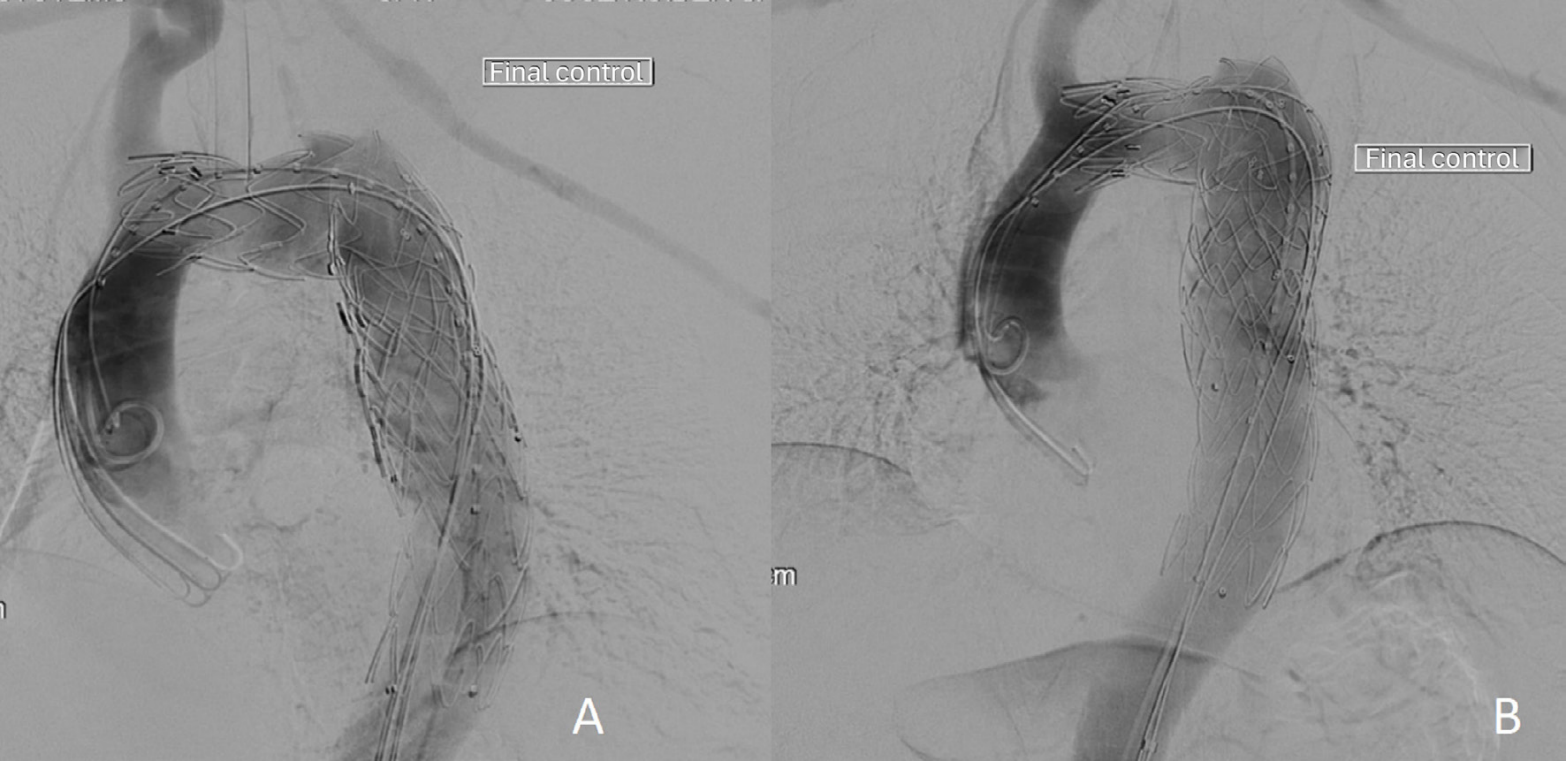
**A** and **B)** Postoperative digital subtraction aortography images showing two endograft devices Valiant^®^ (Medtronic) (38 x 38 x 150 mm and 40 x 40 x 112 mm) with four stents overlapping*.*

In order to perform the second surgical procedure, the small diameter of the external iliac arteries (6 mm) made it necessary to construct an access by implanting a Dacron vascular conduit anastomosed to the distal abdominal aorta. Via a left retroperitoneal access, an 11 mm straight Dacron graft was anastomosed end-to-side to the distal infrarenal abdominal aorta, close above the bifurcation of the iliac arteries (Figure 3). After the access had been constructed, a Lunderquist guidewire was inserted via the conduit, protected by a pigtail catheter. The stent graft device was advanced upwards with radioscopic guidance until, arriving at the aortic arch, support proved insufficient. An attempt was therefore made to advance a stiffer, 260cm guidewire and a 5fr VERT catheter with a fresh introducer via the conduit, but there was still insufficient support for the device. In response, a second stiff guidewire (Lunderquist) was inserted into the introducer to position the stent graft correctly, because of the accentuated angulation of the arch. A 38 x 38 x 150 mm endograft device Valiant^®^ (Medtronic) was deployed into the aortic arch, immediately distal of the brachiocephalic artery. Next, a second Valiant^®^ (Medtronic) endograft device (40 x 40 x 112 mm) was deployed with an overlap of four stents (Figure 4). At the end of the procedure, the conduit was ligated proximally with 2-0 swaged cotton suture and cardiac tape, followed by inspection of hemostasis and closure of the incisions.

**Figure 5:**
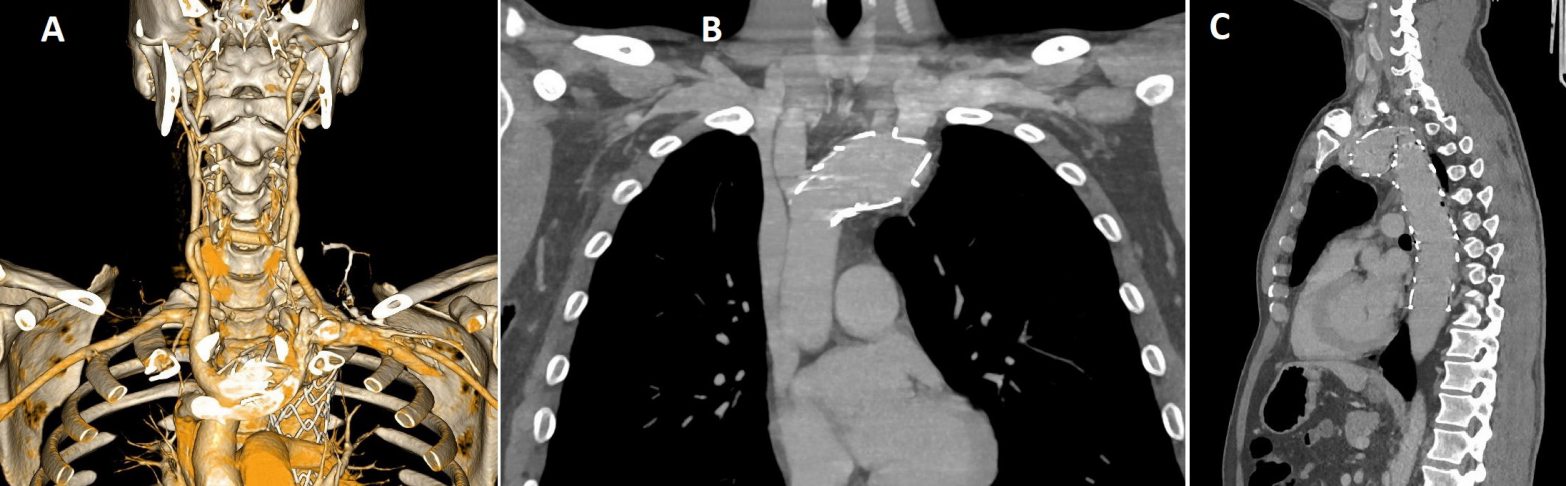
**A)** 3D reconstruction of 1-year postoperative angiotomography (arterial phase), showing patent carotid-carotid and left carotid-subclavian debranching. **B)** Coronal angiotomography image in venous phase (MIP 3 mm), showing the proximal landing zone at 1 year postoperative, with patent brachiocephalic trunk (free flowing) and no endoleaks. **C)** Sagittal angiotomography (venous phase), showing the distal landing zone at 1 year postoperative with no endoleaks.

During the postoperative period, the patient developed a respiratory tract infection, initially treated with cefuroxime and vancomycin and then with piperacillin-tazobactam and rifampicin. He was discharged from hospital on the 12th postoperative day, with explanations about the procedure and control angiotomography ordered for the first outpatients consultation. Angiotomography conducted for 1-year follow-up showed the cervical bypasses were patent and there was no sign of endoleaks (Figure 5).

## DISCUSSION

Diseases of the aortic arch constitute a therapeutic challenge for endovascular repair because of the curvature of the arch and the proximity of the supra-aortic trunks. In the case reported herein, in addition to the difficulties inherent to the anatomic characteristics of the aortic arch, the patient had iliac and femoral arteries with narrow calibers. This situation made it necessary to construct a prosthetic vascular conduit to the distal abdominal aorta to obtain access for the device, which was accomplished with good results.

In the majority of cases, aneurysms of the thoracic aorta are asymptomatic, including those involving the aortic arch. However, some patients may complain of pain, as in the case described, which may indicate that complications are imminent.^3^ Treatment options include conventional or open surgery and endovascular techniques.^3^ The literature highlights the reduced morbidity associated with endovascular treatment of aortic aneurysms, since it is less invasive, the fact that neither hypothermia or extracorporeal circulation are needed, and the good results over the short and medium term.^3^ However, the anatomy of the aortic arch introduces additional complexity to the procedure, such as the importance of maintaining the patency of supra-aortic trunks, which is true of both open and endovascular methods. Revascularization of the supra-aortic trunks can be accomplished using a hybrid procedure, constructing bypasses between the carotid arteries and the left subclavian artery before implanting the endograft. These bypasses, known as cervical “debranching”, enable the endografts to be deployed in zone 1 of the aortic arch.^5^ In the case reported, in addition to requiring extra-anatomic transposition of the supra-aortic trunks, a prosthetic vascular conduit was also constructed to provide access for the stent graft.

Treatment of aneurysms of the aortic arch using hybrid or combined surgery is described in the literature, with good results in selected cases.^6^ One study analyzed 13 years’ experience (1999-2013) of treatment of 179 cases involving the aortic arch using hybrid surgery.^7^ The majority of the patients were male (128) and mean age was 70.2 years. These authors reported a technical success rate of 90.5% (162 dos 179 cases), two deaths, and 15 cases of type 1 endoleak.^7^ Clinical success at 30 days was achieved in 161 cases (89.9%), with a 4.5% mortality rate (8 of the 179 patients). Over the medium term, with a mean follow-up of 27.3±15.7 months, 92.2% of cases (165 of the 179 patients) attained clinical success.^7^ In addition to hybrid surgery, including supra-aortic debranching, completely endovascular treatments have also been described in the literature.^8,9^ One study documented three cases of aortic arch disease treated successfully using endografts with three branches for the supra-aortic trunks.^8^ Other authors have described totally endovascular repair of a saccular aortic arch aneurysm using a stent graft modified with a double fenestration by the surgeon.^9^

Endovascular repair of aneurysms of the thoracic aorta (TEVAR) and of aneurysms of the aortic arch has been increasingly employed for management of complex aortic disease, because of its reduced invasivity and morbidity.^10^ Over the long term, the literature reports good results for endovascular treatment of the thoracic aorta, with survival of 79.3% (95% confidence interval [95%CI] 67.0%-91.7%) over 132 months’ follow-up.^11^ Access via the femoral and iliac vessels introduces an additional challenge to deployment of these devices and one possibility is to construct conduits using synthetic grafts.^12^ In the case described, the patient’s complex anatomy constituted an additional challenge during the procedure, since the aneurysm of the aortic arch was combined with dilation of the subclavian artery, absence of an adequate proximal landing zone, and iliac and femoral arteries with narrow diameters. These anatomic peculiarities made deployment of the stent grafts during the endovascular procedure more difficult. In order to overcome the complexity of the case, in addition to the carotid-carotid-subclavian bypass, a vascular conduit was constructed by end-to-side anastomosis of a Dacron graft above the bifurcation of the iliac arteries. These procedures in conjunction enabled the two stent grafts to be advanced and deployed successfully. Other authors have described a different option for access to the iliac axis for treatment of an abdominal aortic aneurysm: construction of an endoconduit by releasing a covered stent in the common femoral artery and externalizing it, enabling passage of the endovascular grafts.^13^ In the case presented, the option of using a Dacron graft conduit was chosen because the patient’s iliac arteries were of narrow caliber and the aneurysm involved the aortic arch. Although endovascular treatment of aneurysms is considered less invasive and in general demonstrates good results over the short term, complications associated with endovascular repair have been described in the literature. These include endoleaks, stroke, endograft collapse, injuries related to access, and kidney failure.^11,14^ Some authors recommend considering the patient’s individual surgical risk when selecting the treatment option for aneurysms of the aortic arch.^5^

Treatment of the aortic arch involves individualized management, considering the characteristics of the patient (anatomy, comorbidities, and risks) and of the aneurysm (location and dimensions). In the case presented, the patient’s complex anatomy demanded endovascular treatment in conjunction with construction of a prosthetic vascular conduit, enabling passage of the devices and deployment of the two endografts. Despite some difficulties encountered during the procedure, the results were satisfactory, with exclusion of the aneurysm. Postoperative follow-up is essential for long-term success of endovascular treatment of aneurysms of the aortic arch.
