# Supplementary material for: Treatment of aortic arch and left subclavian artery aneurysms in two stages using combined surgical approaches: case report
Source: J Vasc Bras. 2025 Apr 7;24:e20240127. doi: 10.1590/1677-5449.202401272 (PMC11991671; doi:10.1590/1677-5449.202401272)
Supplement: Translation

# Translation: Tratamento de aneurismas de arco aórtico e artéria subclávia esquerda em dois tempos por vias combinadas: relato de caso

**DOI:** 10.1590/1677-5449.202401271

**Authors:** Josué Song Der Wu, Lucas Porto Maurity Dias, Giuliano Giová Volpiani, Valter Castelli, Vanessa Prado dos Santos, Luiz Eduardo Meucci Pereira Nogueira, Walkíria Hueb Bernardi, Roberto Augusto Caffaro

**Affiliations:** 1 Santa Casa de São Paulo, Faculdade de Ciências Médicas, São Paulo, SP, Brasil.; 2 Irmandade da Santa Casa de Misericórdia de São Paulo – ISCMSP, São Paulo, SP, Brasil.; 3 Universidade Federal da Bahia – UFBA, Faculdade de Medicina da Bahia, Salvador, BA, Brasil.

**Keywords:** arco aórtico, correção endovascular de aneurisma, procedimentos cirúrgicos vasculares

## Abstract

O aneurisma do arco aórtico é geralmente assintomático, sendo diagnosticado incidentalmente através de exames como tomografia computadorizada, ressonância magnética ou ecocardiograma. O tratamento convencional envolve cirurgia por toracotomia, também podendo ser realizado o tratamento através da técnica endovascular. O artigo apresenta um relato de caso de um aneurisma complexo do arco aórtico com comprometimento da artéria subclávia esquerda. O tratamento foi iniciado através de uma derivação dos troncos supra-aórticos, carotídeo-carotídea e carotídeo-subclávia, seguida da ligadura das artérias carótida comum e subclávia esquerda. Em um segundo momento, foi necessária a confecção de um conduíte vascular cirúrgico com prótese de Dacron para acesso ao dispositivo de entrega da endoprótese no arco aórtico, devido ao calibre reduzido das artérias femorais e ilíacas externas. O caso destaca a evolução satisfatória de um caso de aneurisma do arco aórtico de anatomia complexa.

## INTRODUÇÃO

No mundo, os aneurismas da aorta estiveram relacionados ao óbito de 172.427 pessoas em 2019, representando um aumento de 82,1% em relação a 1990 (94.698 óbitos)^1^ . Apesar do aumento em números absolutos, a mortalidade padronizada por idade devido a aneurismas da aorta por 100.000 habitantes apresentou uma tendência decrescente (17,9%), passando de 2,70/100.000 habitantes em 1990 para 2,21/100.000 habitantes em 2019^1^ .

A doença aneurismática da aorta é definida como uma dilatação localizada que envolve todas as camadas do vaso, sendo a aorta abdominal o segmento mais comumente acometido^2^ . Os fatores associados a maior risco de crescimento dos aneurismas da aorta incluem idade, sexo feminino, doença pulmonar obstrutiva crônica, hipertensão arterial, história familiar e o diâmetro do aneurisma^3^ . A literatura aponta que o diâmetro inicial do aneurisma influencia sua taxa de crescimento, sendo que aneurismas maiores crescem mais rapidamente^3^ . Os aneurismas do arco aórtico são raros, geralmente assintomáticos e representam um desafio no diagnóstico e tratamento^2^ . A importância dos troncos supra-aórticos, de seus principais ramos e da convexidade do arco aórtico adiciona uma complexidade significativa às opções terapêuticas nas doenças do arco aórtico^2^ .

Os aneurismas de aorta torácica e do arco aórtico apresentam como história natural o aumento progressivo do diâmetro, devendo ser acompanhados por meio de métodos de imagem para indicação cirúrgica. As diretrizes da American Heart Association sugerem que diversos fatores sejam considerados para a indicação cirúrgica dos aneurismas da aorta torácica, incluindo o diâmetro, o padrão de crescimento, a avaliação detalhada do caso, o diagnóstico da doença associada à dilatação da aorta, como a síndrome de Marfan, a experiência da equipe e a medida proporcional da aorta, levando em consideração a altura do paciente^4^ . O tratamento cirúrgico dos aneurismas de aorta torácica pode ser realizado por cirurgia convencional ou pela técnica endovascular. O acesso ao arco aórtico na cirurgia convencional requer uma esternotomia mediana para substituição da porção dilatada da aorta por uma prótese vascular^3^ . A segunda técnica, a endovascular, é menos invasiva e conta com o auxílio de fluoroscopia para a realização do procedimento, que é feito por meio de dissecção ou punção bilateral das artérias femorais comuns, navegação por fios-guia e implante de uma endoprótese para revestir internamente a aorta aneurismática, excluindo o aneurisma. Para o acesso do dispositivo da endoprótese, a técnica geralmente requer que as femorais e ilíacas tenham um calibre mínimo de 8 mm. Considerando que o reparo convencional dos aneurismas do arco aórtico necessita da interrupção do fluxo para os troncos supra-aórticos, a técnica endovascular vem apresentando novas soluções e alternativas para o tratamento das doenças do arco aórtico^3^ .

Nas doenças da aorta, as decisões importantes sobre a indicação de intervenção, o tipo de reparo cirúrgico e a seleção entre opções cirúrgicas abertas e/ou endovasculares precisam ser minuciosamente estudadas e compartilhadas^4^ . Neste relato de caso, apresentamos a correção endovascular de um aneurisma do arco aórtico, que exigiu a confecção cirúrgica de um conduíte de prótese de Dacron para viabilizar o acesso do dispositivo. O estudo foi aprovado pelo Comitê de Ética em Pesquisa da Santa Casa de Misericórdia de São Paulo (Certificado de Apresentação de Apreciação Ética: 80949724.9.0000.5479; número do parecer: 6.978.599).

## DESCRIÇÃO DO CASO

Homem de 43 anos, natural e procedente da Bolívia, hipertenso e tabagista (25 anos-maço), foi admitido na unidade de emergência da Santa Casa de Misericórdia de São Paulo com quadro de dor torácica retroesternal que irradiava para o dorso há cerca de 1 ano. Devido à piora da dor nos últimos dias, procurou atendimento médico. Relata que, há 1 ano, durante os exames de rotina do trabalho, realizou um raio X de tórax que evidenciou alargamento do mediastino, sendo então investigado e diagnosticado com doença aneurismática da aorta. Não apresentava alterações ao exame físico.

A angiotomografia de aorta mostrou um volumoso aneurisma na origem da artéria subclávia esquerda e na porção distal da crossa da aorta (Figura 1). A artéria subclávia apresentava um diâmetro transverso máximo de 4,2 x 4,1 cm, com extensão de aproximadamente 4,2 cm (Figura 2). O paciente encontrava-se assintomático, e o exame de imagem contrastado não evidenciou sinais de rotura ou iminência de rotura, sendo, portanto, optado pela correção cirúrgica do aneurisma de maneira programada e estagiada. A análise detalhada das medidas e da anatomia das dilatações aneurismáticas revelou a necessidade de ligadura das artérias carótida comum e subclávia esquerdas para obtenção de um colo proximal adequado à fixação da endoprótese. Sendo assim, foi realizada uma ponte derivação carotídeo-carotídea e carotídeo-subclávia esquerda com politetrafluoretileno (PTFE) expandido aramado (6 mm), para posterior reparo endovascular do aneurisma do arco aórtico. O intervalo entre os dois procedimentos foi de 20 dias, e ambos transcorreram sem intercorrências.

**Figura 1:** **A** e **B)** Imagens dos cortes transversais da angiotomografia pré-operatória da aorta, evidenciando aneurisma do arco aórtico com comprometimento da artéria subclávia esquerda, com diâmetro transverso máximo de 4,2 x 4,1 cm.

**Figura 2:** **A)** Imagem da angiografia de subtração digital e **B** e **C)** da angiotomografia, mostrando dilatação aneurismática do arco aórtico e da artéria subclávia esquerda (seta).

Na primeira cirurgia, foi realizada uma cervicotomia longitudinal direita para acessar a artéria carótida comum, procedimento que também foi replicado no lado esquerdo. Adicionalmente, uma incisão supraclavicular oblíqua à esquerda foi feita para identificar e isolar a artéria subclávia esquerda, que se mostrava bastante dilatada. Procedeu-se a uma derivação carotídeo-carotídea e carotídeo-subclávia esquerda utilizando uma prótese de PTFE com 6 mm de diâmetro, para manter o fluxo subclávio e carotídeo esquerdos. Na sequência, foram realizadas ligaduras proximais na artéria subclávia esquerda e na carótida comum esquerda, evitando o reenchimento do saco aneurismático por deflúvio e prevenindo a ocorrência posterior de vazamento (*endoleak* tipo 2). Por fim, o local cirúrgico foi revisado para garantir a hemostasia, drenado e suturado.

Para a realização da segunda etapa do procedimento cirúrgico, foi necessária a confecção de um acesso através do implante de um conduíte vascular de Dacron anastomosado à aorta abdominal distal, devido ao diâmetro reduzido das artérias ilíacas externas (6 mm). Através de um acesso retroperitoneal esquerdo, realizou-se uma anastomose terminolateral de um conduíte de Dacron reto de 11 mm em aorta abdominal infrarrenal distal, logo acima da bifurcação das artérias ilíacas (Figura 3). Após a confecção do acesso, um fio-guia Lunderquist foi introduzido pelo conduíte, protegido por um cateter Pigtail. A subida da endoprótese foi guiada por radioscopia; contudo, ao chegar à crossa da aorta, o suporte não estava adequado. Optou-se pela passagem de fio-guia com maior rigidez (*stiff*) de 260 cm e de cateter vert 5fr em novo introdutor através do conduíte, cursando também com suporte insuficiente para o dispositivo. Diante disso, foi introduzido um segundo fio-guia rígido (Lunderquist) no introdutor para posicionar adequadamente a endoprótese, devido à acentuada angulação da crossa da aorta. Foi implantada uma endoprótese Valiant^®^ (Medtronic) de 38 x 38 x 150 mm no arco da aorta, imediatamente distal à artéria braquiocefálica. Em seguida, implantou-se uma segunda endoprótese Valiant^®^ 40 x 40 x 112 mm, com uma sobreposição (*overlap*) de quatro *stents* (Figura 4). Ao término do procedimento, foram feitas a ligadura proximal do conduíte com algodão 2-0 agulhado e fita cardíaca, a revisão da hemostasia e a síntese das incisões.

**Figura 3:** **A)** Imagem da angiografia do território aortoilíaco. **B)** Imagem intraoperatória da anastomose terminolateral de conduíte vascular com prótese de Dacron reta de 11 mm na aorta infrarrenal para o acesso retroperitoneal esquerdo ao dispositivo. **C)** Arteriografia do conduíte para o acesso do dispositivo da endoprótese ao arco aórtico.

**Figura 4:** **A** e **B)** Imagens da aortografia de subtração digital pós-operatória, mostrando a sobreposição das duas endopróteses Valiant^®^ (38 x 38 x 150 mm e 40 x 40 x 112 mm) com sobreposição de quatro *stents.*

No pós-operatório, o paciente evoluiu com uma infecção do trato respiratório, inicialmente tratada com cefuroxima e vancomicina e, posteriormente, com piperacilina-tazobactam e rifampicina. Recebeu alta hospitalar no 12º dia de pós-operatório, com orientações sobre o procedimento e solicitação de angiotomografia de controle para o próximo retorno ambulatorial. Na angiotomografia realizada no seguimento de 1 ano, as derivações cervicais encontravam-se pérvias e não havia sinal de *endoleaks* (Figura 5).

**Figura 5:** **A)** Angiotomografia com reconstrução 3D (fase arterial) pós-operatória de 1 ano, demonstrando enxertos carotídeo-carotídeo e carotídeo-subclávia esquerda pérvios. **B)** Angiotomografia com corte coronal em fase venosa (MIP 3 mm), mostrando o colo proximal no pós-operatório de 1 ano, com tronco braquiocefálico pérvio (*free-flow*) e ausência de *endoleak*. **C)** Angiotomografia com corte sagital (fase venosa), mostrando o colo distal no pós-operatório de 1 ano e ausência de *endoleak*.

## DISCUSSÃO

As doenças do arco aórtico representam um desafio terapêutico para o reparo endovascular devido à curvatura do arco e à proximidade dos troncos supra-aórticos. No caso apresentado, além das dificuldades inerentes às características anatômicas do arco aórtico, o paciente apresentava artérias ilíacas e femorais de calibre reduzido. Essa condição exigiu a confecção de um conduíte vascular protético na aorta abdominal distal para permitir o acesso do dispositivo, com bom resultado.

Na maioria dos casos, os aneurismas da aorta torácica, incluindo os do arco aórtico, são assintomáticos. No entanto, alguns pacientes possam apresentar dor, como no caso descrito, o que pode indicar a iminência de complicações^3^ . As opções terapêuticas incluem a cirurgia convencional ou aberta e a técnica endovascular^3^ . A literatura destaca uma menor morbidade associada ao tratamento endovascular dos aneurismas da aorta, por ser menos invasivo, dispensar hipotermia ou circulação extracorpórea e apresentar bons resultados a curto e médio prazos^3^ . Apesar disso, a anatomia do arco aórtico adiciona complexidade ao procedimento, como a importância de manter a perviedade dos troncos supra-aórticos tanto em técnicas abertas quanto endovasculares. A revascularização dos troncos supra-aórticos pode ser realizada através de um procedimento híbrido, com a confecção de derivações entre as artérias carótidas e subclávia esquerda antes do implante da endoprótese. Essas derivações, conhecidas como “*debranching*” cervical, permitem o implante de endopróteses na zona 1 do arco aórtico^5^ . No caso apresentado, além da necessidade de derivação extra-anatômica dos troncos supra-aórticos, foi confeccionado um conduíte vascular protético para possibilitar o acesso da endoprótese.

O tratamento de aneurismas do arco aórtico por meio de cirurgias híbridas ou combinadas é descrito na literatura, com bons resultados em casos selecionados^6^ . Um estudo avaliou uma experiência de 13 anos (1999-2013) no tratamento de 179 casos utilizando cirurgias híbridas envolvendo o arco aórtico^7^ . A maioria dos pacientes era do sexo masculino (128), com média de idade de 70,2 anos. Os autores relataram uma taxa de sucesso técnico de 90,5% (162 dos 179 casos), dois óbitos e 15 casos de vazamento, ou "*endoleaks*", do tipo 1^7^ . Foi registrado sucesso clínico em 30 dias para 161 casos (89,9%), com taxa de mortalidade de 4,5% (8 dos 179 pacientes). A médio prazo, com seguimento médio de 27,3±15,7 meses, 92,2% dos casos (165 dos 179 pacientes) apresentaram sucesso clínico^7^ . Além das cirurgias híbridas, com a realização de derivações ou “*debranching*” dos troncos supra-aórticos, tratamentos completamente endovasculares vêm sendo descritos na literatura^8,9^ . Um estudo documentou três casos de patologias do arco aórtico tratados com sucesso utilizando endopróteses com três ramificações para os troncos supra-aórticos^8^ . Outros autores relataram o reparo totalmente endovascular de um aneurisma sacular do arco aórtico, realizado com o implante de endoprótese com dupla fenestra modificada pelo cirurgião^9^ .

O tratamento endovascular dos aneurismas da aorta torácica (TEVAR) e dos aneurismas do arco aórtico tem sido cada vez mais empregado no manejo de doenças complexas da aorta, devido ao seu caráter menos invasivo e à menor morbidade^10^ . A longo prazo, a literatura aponta bons resultados para o tratamento endovascular da aorta torácica, com sobrevida de 79,3% (intervalo de confiança de 95% [IC95%] 67,0%-91,7%) em um seguimento de 132 meses^11^ . O acesso pelos vasos femorais e ilíacos representa um desafio adicional para o implante desses dispositivos, havendo a possibilidade de confecção de conduítes com próteses sintéticas^12^ . No caso apresentado, a anatomia complexa do paciente se apresentou como um desafio adicional durante o procedimento, pela presença de um aneurisma de arco aórtico associado à dilatação da artéria subclávia, sem um colo proximal adequado e com diâmetro reduzido das artérias ilíacas e femorais. Essas particularidades anatômicas dificultaram a implantação das endopróteses no procedimento endovascular. Para superar a complexidade do caso, foi realizada, além da derivação carotídeo-carotídeo-subclávio, a confecção de um conduíte vascular com prótese de Dacron, com anastomose terminolateral acima da bifurcação das artérias ilíacas. O conjunto de procedimentos permitiu a passagem e o implante adequado das duas endopróteses, com sucesso. No tratamento de um aneurisma da aorta abdominal, alguns autores descreveram uma opção diferente para o acesso ao eixo ilíaco: a confecção de um endoconduto através da liberação e exteriorização de um *stent* recoberto na artéria femoral comum, viabilizando a passagem das endopróteses^13^ . No caso apresentado, optou-se pela utilização de um conduíte com prótese de Dacron, por se tratar de paciente com artérias ilíacas de calibre reduzido e aneurisma do arco aórtico. Apesar de o tratamento endovascular dos aneurismas ser considerado menos invasivo e apresentar, em geral, boa evolução a curto prazo, complicações associadas ao reparo endovascular estão descritas na literatura. Entre elas, destacam-se vazamentos ou *endoleaks*, acidente vascular cerebral, colapso da endoprótese, lesões relacionadas ao acesso e insuficiência renal^11,14^ . Alguns autores recomendam considerar o risco cirúrgico individual do paciente na seleção da opção terapêutica dos aneurismas do arco aórtico^5^ .

O tratamento do arco aórtico envolve uma abordagem individualizada, considerando as características do paciente (anatomia, comorbidades e riscos) e do aneurisma (localização e dimensões). No caso apresentado, a anatomia complexa do paciente exigiu um tratamento endovascular associado à confecção de um conduíte vascular protético, que permitiu a passagem adequada dos dispositivos e o implante das duas endopróteses. Apesar das dificuldades encontradas durante o procedimento, o resultado foi satisfatório, com exclusão do aneurisma. O acompanhamento pós-operatório é fundamental para o sucesso a longo prazo do tratamento endovascular dos aneurismas do arco aórtico.
